# Supplementary material for: Archaeal lipid biomarker constraints on the Paleocene-Eocene carbon isotope excursion
Source: Nat Commun. 2019 Oct 4;10:4519. doi: 10.1038/s41467-019-12553-3 (PMC6778145; doi:10.1038/s41467-019-12553-3)
Supplement: Supplementary file 1 — Supplementary Information [file 41467_2019_12553_MOESM1_ESM.pdf]

Supplementary Information

Archaeal lipid biomarker constraints on the Paleocene-Eocene carbon isotope excursion

Elling *et al.*

Supplementary Figures

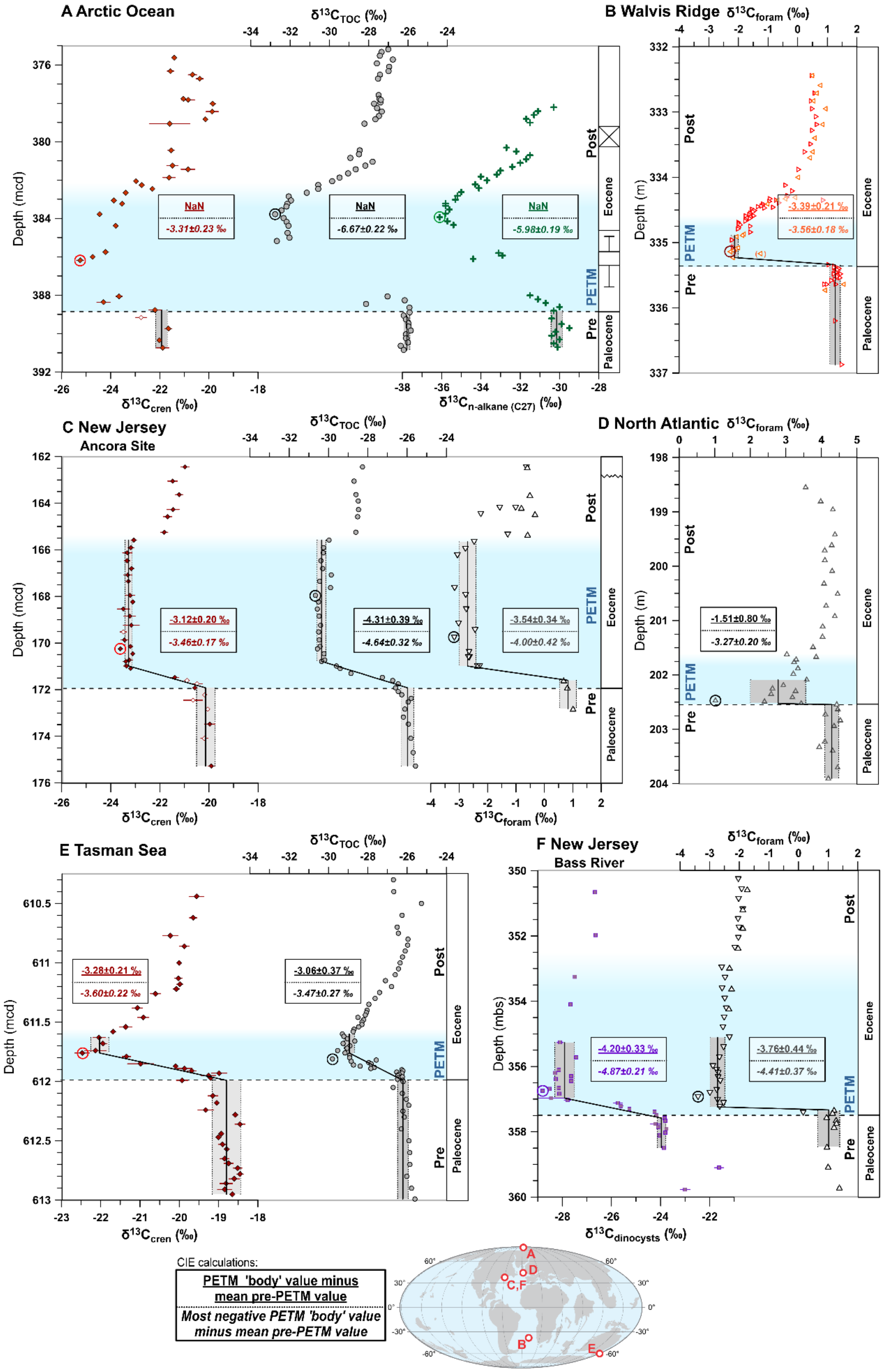

Supplementary Figure 1. Magnitude determination for the Paleocene-Eocene carbon isotope excursion recorded in crenarchaeol and other substrates. Stable carbon isotope records from (**A**) the Arctic Ocean based on crenarchaeol ($\delta^{13}C_{cren}$; this study), total organic carbon ($\delta^{13}C_{TOC}$; this study), and leaf-waxes ($\delta^{13}C_{n\text{-}alkanes}$ (C_{27}); ref. [1]), from (**B**) ODP Site 1263 at Walvis Ridge based on benthic foraminifera ($\delta^{13}C_{foram}$; *Nuttallides truempyi*: left-pointed triangle, and *Oridorsalis umbonatus*: right-pointed triangle; ref. [2]), from (**C**) the New Jersey Shelf (Ancora site) based on crenarchaeol (this study), total organic carbon (this study), benthic foraminifera (*Anomalinoides acutus*: reversed triangle, and *Cibicidoides* spp.: triangle; $\delta^{13}C_{foram}$; ref. [3]), and bulk carbonates ($\delta^{13}C_{carbonate}$; ref. [4]), from (**D**) the North Atlantic (Bay of Biscay) based on planktic foraminifera (*Morozovella subbotinae*; $\delta^{13}C_{foram}$; ref. [5]), from (**E**) Tasman Sea sediment cores based on crenarchaeol (this study) and total organic carbon (ref. [6]), and from (**F**) the New Jersey Shelf (Bass River site) based on bulk dinocyst organic matter ($\delta^{13}C_{dinocysts}$; ref. [7]) and based on benthic foraminifera (as in (**C**), ref. [8]). We estimate the CIE based on two methods: first, as difference between mean pre and peak-PETM values (bold and underlined; with the CIE error representing the propagated uncertainties of the means, i.e., their 1σ standard deviations), and second, as difference between the pre-PETM $\delta^{13}C_{cren}$ mean and the lowest $\delta^{13}C_{cren}$ value during the PETM (italicized; with the CIE error representing the propagated uncertainty of the 1σ standard deviation of the pre-PETM mean and the reported/typical error of the PETM minimum value). For the latter approach, we highlighted the minimum PETM value of each record with a circle. The intervals of the pre- and peak-PETM were determined through ramp function fitting[9]. Please note that although this approach is imperfect (through an a priori assumption of the character of PETM transition (a ramp) and the dependence of the results on the length of pre-PETM record), it provides an objective way of estimating onset and end of the transition into the PETM, allowing a reproducible determination of the CIE. Open symbols for $\delta^{13}C_{cren}$ in (**A**) and (**B**) represent samples with low signal-to-background ratios (F2/F1<2; see Methods). Error bars of the $\delta^{13}C_{cren}$ data represent one standard deviation of replicate analyses (n=5). Error bars of $\delta^{13}C_{cren}$ are not displayed where they are smaller than the symbol. The onset of the PETM (as indicated by the left-most record in each panel) is highlighted with a stippled line. Error bars in the stratigraphic column of the Arctic Ocean record indicate uncertainty in sample position due to poor core recovery. Core locations for each panel are indicated on the map.

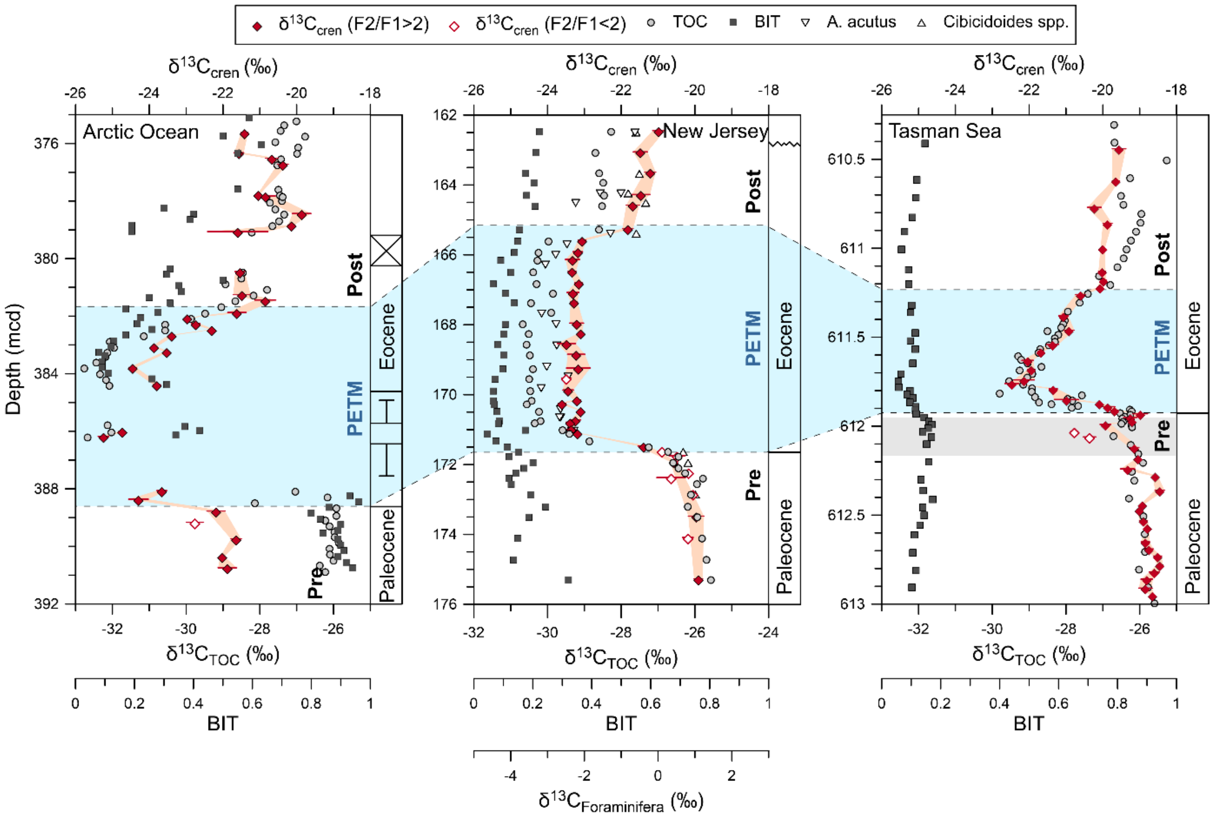

Supplementary Figure 2. Reconstructions of terrigenous input across the Paleocene-Eocene Thermal Maximum. Branched over isoprenoid tetraether index (BIT, grey squares) values at the Arctic Ocean study site (ref. [10]), at the New Jersey site (this study), and in the Tasman Sea (ref. [6]) and our new carbon stable isotopic ratios of crenarchaeol ($\delta^{13}C_{cren}$, red diamonds) are shown alongside stable carbon isotope data of total organic carbon ($\delta^{13}C_{TOC}$; Arctic Ocean, New Jersey: this study; Tasman Sea: ref. [6]) and of the foraminifera *Anomalinoides acutus* and *Cibicidoides* spp. ($\delta^{13}C_{Foraminifera}$; ref. [3]). Open symbols for $\delta^{13}C_{cren}$ represent samples with low signal-to-background ratios (F2/F1<2); low F2/F1 samples tend towards more negative $\delta^{13}C_{cren}$ values (see Methods). Error bars of $\delta^{13}C_{cren}$ represent one standard deviation of replicate analyses (n=5). Error bars of $\delta^{13}C_{cren}$ are not displayed where they are smaller than the symbol. Boundaries of the PETM and pre-onset event are shown in blue and gray shading, respectively.

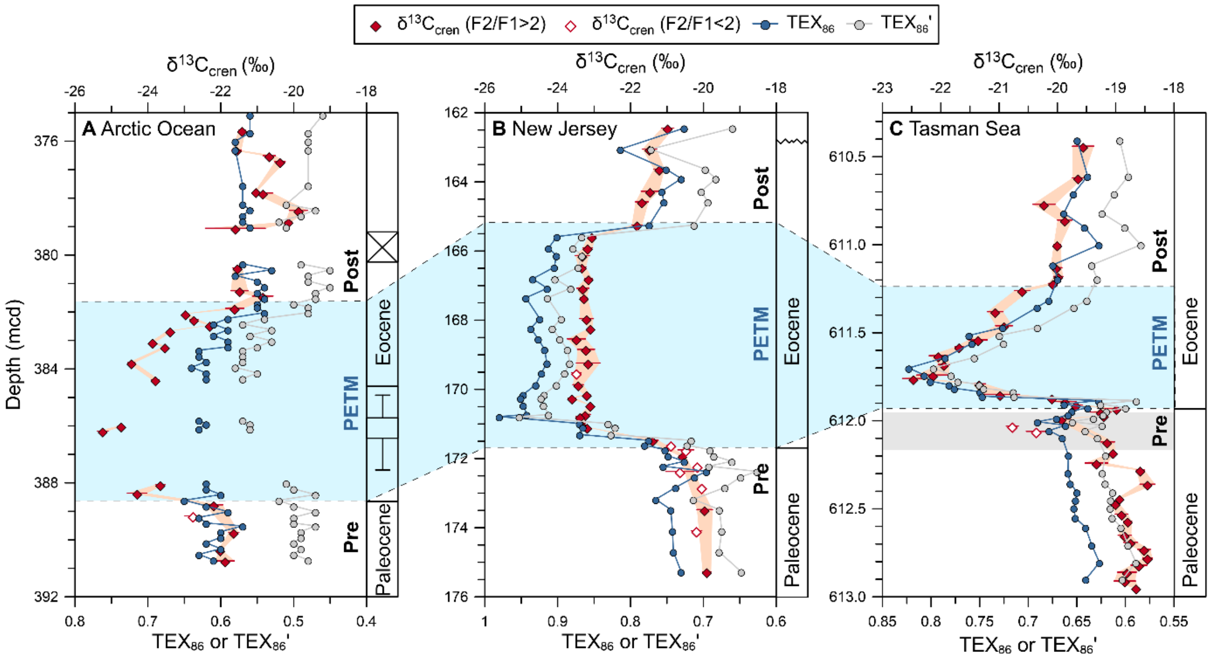

Supplementary Figure 3. Comparison of archaeal lipid-based paleotemperature proxies. TEX_{86} and TEX_{86}' index values (plotted on an inverted axis; Arctic Ocean: ref. [10], New Jersey: this study; Tasman Sea: ref. [6]) and carbon isotopic ratios of crenarchaeol ($\delta^{13}C_{cren}$) during the late Paleocene and early Eocene at three globally distributed sites (**A**, Arctic Ocean; **B**, New Jersey Shelf; **C**, Tasman Sea). Closed and open symbols for $\delta^{13}C_{cren}$ represent samples with high and low signal-to-background ratios (F2/F1), respectively; low F2/F1 samples tend towards more negative $\delta^{13}C_{cren}$ values (see Methods). Error bars of $\delta^{13}C_{cren}$ represent one standard deviation of replicate analyses (n=5). Error bars of $\delta^{13}C_{cren}$ are not displayed where they are smaller than the symbol. Boundaries of the PETM and pre-onset event are shown in blue and gray shading, respectively.

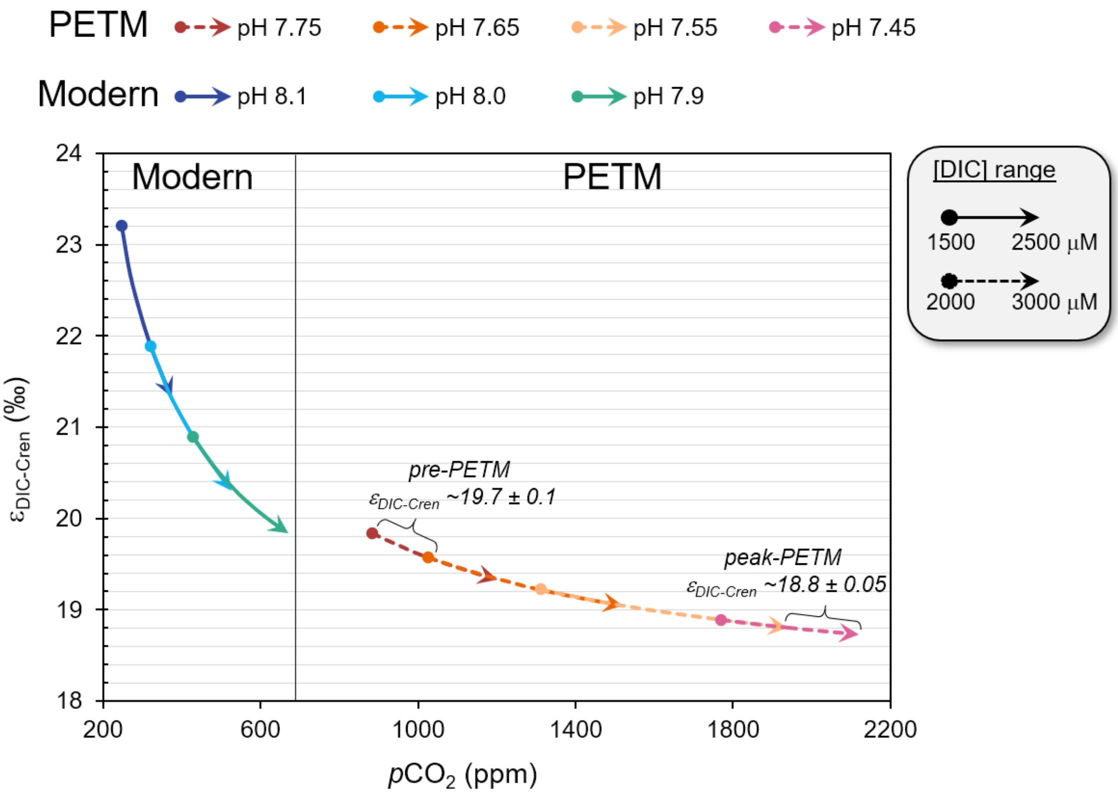

Supplementary Figure 4. Model estimates of archaeal carbon isotope fractionation. Sensitivity of archaeal carbon isotope fractionation ($\varepsilon_{DIC\text{-}Cren}$) to pCO$_2$ (assuming 96 hour doubling time) calculated following Pearson et al.[11] for two scenarios: "Modern" and "PETM". Modern parameters: 15 °C seawater temperature, salinity 35, [DIC] between 1500-2500 µmol kg^{-1}, pH 8.1 to 7.9. PETM parameters[5,12]: 25 °C seawater temperature, salinity 35, [DIC] 2000-3000 µmol kg^{-1}, pH 7.75 to 7.45.

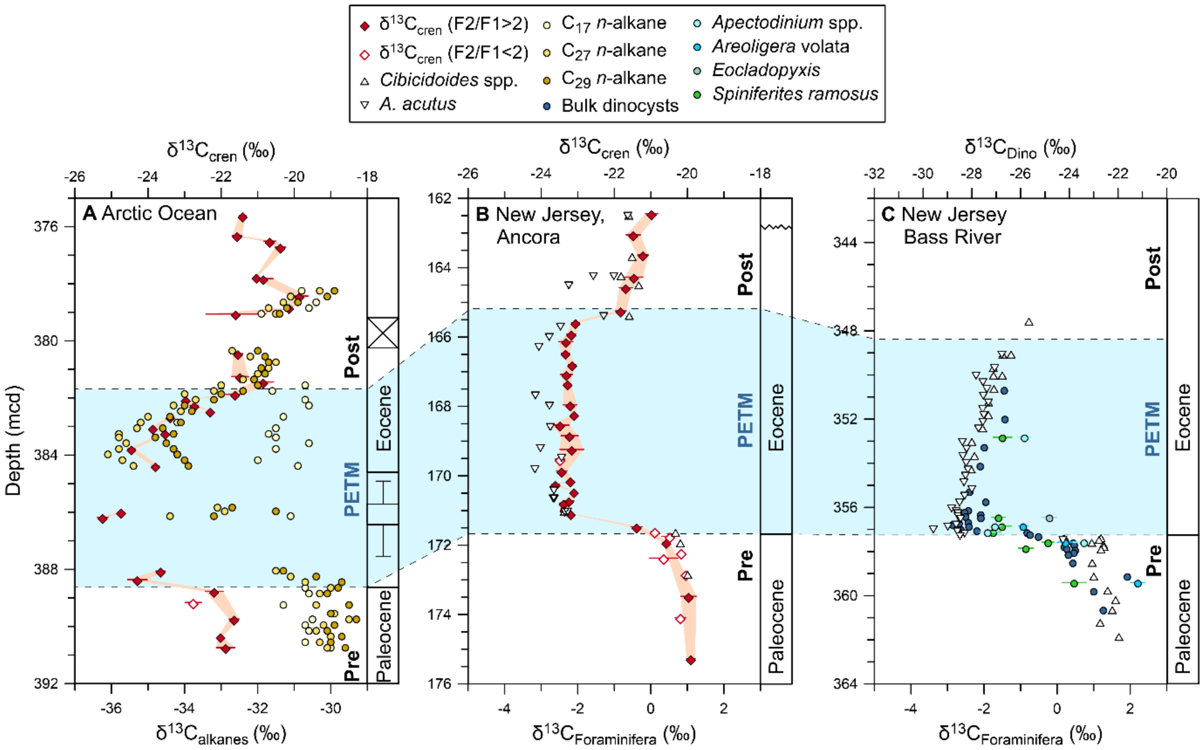

Supplementary Figure 5. Comparison of the Paleocene-Eocene carbon isotope excursion recorded in crenarchaeol with additional marine substrates. Carbon isotopic ratios of crenarchaeol ($\delta^{13}C_{cren}$; this study) and literature data of other marine (foraminifera, dinocysts, C_{24}*n*-alkane) and terrestrial (C_{27}, C_{29}*n*-alkanes) substrates for the Arctic Ocean (**A**; $\delta^{13}C_{alkanes}$; ref. [1]) and the two New Jersey shelf sites Ancora (**B**) and Bass River (**C**; foraminifera *Anomalinoides acutus* and *Cibicidoides* spp., $\delta^{13}C_{Foraminifera}$; ref. [3]; dinocysts *Apectodinium* spp., *Areoligeravolata*, *Eocladopyxis*, and *Spiniferites ramosus* as well as bulk dinocyst data, $\delta^{13}C_{Dino}$; refs. [7,13]). Closed and open symbols for $\delta^{13}C_{cren}$ represent samples with high and low signal-to-background ratios (F2/F1), respectively; low F2/F1 samples tend towards more negative $\delta^{13}C_{cren}$ values (see Methods). Error bars for $\delta^{13}C_{cren}$ represent one standard deviation of replicate analyses (n=5). Error bars for $\delta^{13}C_{Dino}$ represent standard errors of replicate analyses (n=14-70; ref. [13]). Error bars of $\delta^{13}C_{cren}$ and $\delta^{13}C_{Dino}$ are not displayed where they are smaller than the symbol. Boundaries of the PETM are shown in blue (based on refs. [10,14]). Error bars in the stratigraphic column of the Arctic Ocean record indicate uncertainty in sample position due to poor core recovery.

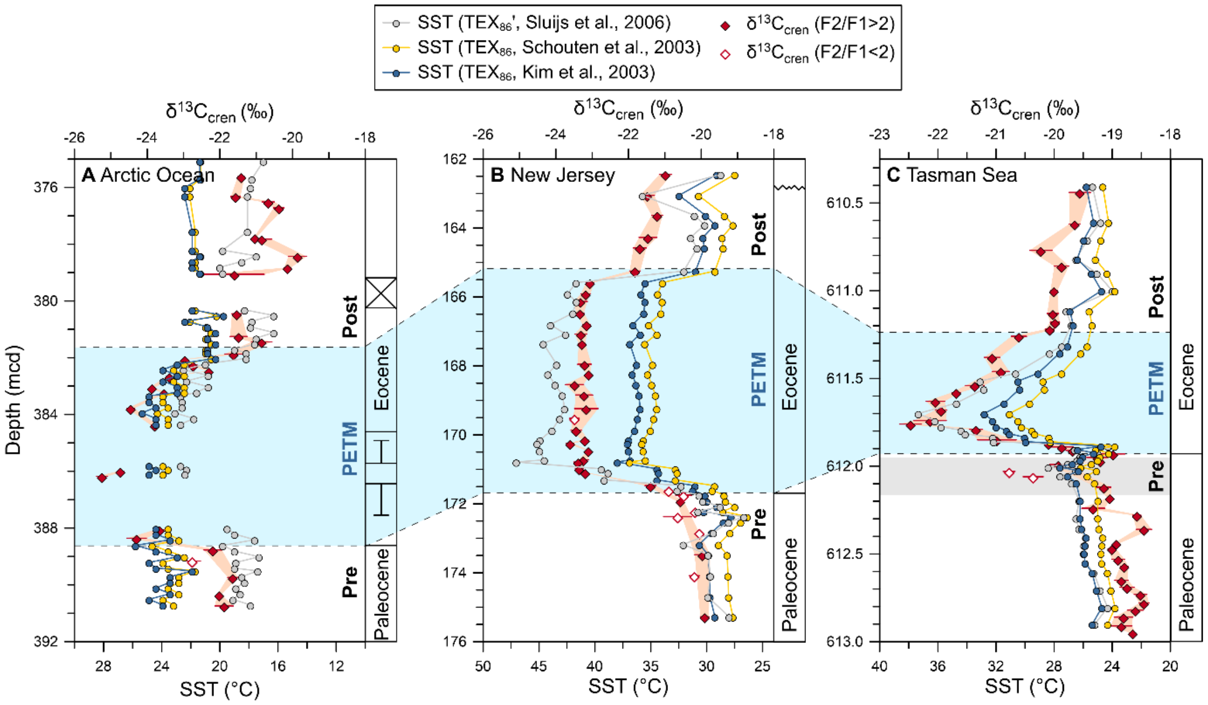

Supplementary Figure 6. Comparison of paleotemperature calibrations. Sea surface temperatures (SST) reconstructed through a number of calibrations[10,15,16] of the TEX_{86} and TEX_{86}' index values in the Arctic Ocean[10] (**A**), at our New Jersey study site (**B**, this study) and in the Tasman Sea[6] (**C**; all data are shown on an inverted axis), as well as our new stable carbon isotope data of crenarchaeol ($\delta^{13}C_{cren}$) at our Arctic Ocean site (**A**), at the New Jersey Shelf (**B**), and in the Tasman Sea (**C**). Closed and open symbols for $\delta^{13}C_{cren}$ represent samples with high and low signal-to-background ratios (F2/F1), respectively; low F2/F1 samples tend towards more negative $\delta^{13}C_{cren}$ values (see Methods). Error bars for $\delta^{13}C_{cren}$ represent one standard deviation of replicate analyses (n=5). Error bars of $\delta^{13}C_{cren}$ are not displayed where they are smaller than the symbol. Boundaries of the PETM and pre-onset event are shown in blue and gray shading, respectively.

Supplementary References

1. Pagani, M. *et al.* Arctic hydrology during global warming at the Palaeocene/Eocene thermal maximum. *Nature* **442**, 671–675 (2006).
2. McCarren, H., Thomas, E., Hasegawa, T., Röhl, U. & Zachos, J. C. Depth dependency of the Paleocene-Eocene carbon isotope excursion: Paired benthic and terrestrial biomarker records (Ocean Drilling Program Leg 208, Walvis Ridge). *Geochemistry, Geophysics, Geosystems* **9**, Q10008 (2008).
3. Cramer, B. S. & Kent, D. V. Bolide summer: The Paleocene/Eocene thermal maximum as a response to an extraterrestrial trigger. *Palaeogeography, Palaeoclimatology, Palaeoecology* **224**, 144–166 (2005).
4. Stassen, P., Thomas, E. & Speijer, R. P. Integrated stratigraphy of the Paleocene-Eocene thermal maximum in the New Jersey Coastal Plain: Toward understanding the effects of global warming in a shelf environment. *Paleoceanography* **27**, PA4210 (2012).
5. Gutjahr, M. *et al.* Very large release of mostly volcanic carbon during the Palaeocene–Eocene Thermal Maximum. *Nature* **548**, 573–577 (2017).
6. Sluijs, A. *et al.* Southern ocean warming, sea level and hydrological change during the Paleocene-Eocene thermal maximum. *Climate of the Past* **7**, 47–61 (2011).
7. Sluijs, A. *et al.* Environmental precursors to rapid light carbon injection at the Palaeocene/Eocene boundary. *Nature* **450**, 1218–1221 (2007).
8. John, C. M. *et al.* North American continental margin records of the Paleocene-Eocene thermal maximum: Implications for global carbon and hydrological cycling. *Paleoceanography* **23**, PA2217 (2008).
9. Mudelsee, M. Ramp function regression: a tool for quantifying climate transitions. *Computers & Geosciences* **26**, 293–307 (2000).

10. Sluijs, A. *et al.* Subtropical Arctic Ocean temperatures during the Palaeocene/Eocene thermal maximum. *Nature* **441**, 610–613 (2006).

11. Pearson, A., Hurley, S. J., Elling, F. J. & Wilkes, E. B. CO_2-dependent carbon isotope fractionation in Archaea, Part I: Modeling the 3HP/4HB pathway. *Geochimica et Cosmochimica Acta* **261**, 368–382 (2019).

12. Zeebe, R. E., Zachos, J. C. & Dickens, G. R. Carbon dioxide forcing alone insufficient to explain Palaeocene–Eocene Thermal Maximum warming. *Nature Geoscience* **2**, 576–580 (2009).

13. Sluijs, A., van Roij, L., Frieling, J., Laks, J. & Reichart, G.-J. Single-species dinoflagellate cyst carbon isotope ecology across the Paleocene-Eocene Thermal Maximum. *Geology* **46**, 79–82 (2018).

14. Stassen, P., Thomas, E. & Speijer, R. P. Integrated stratigraphy of the Paleocene-Eocene thermal maximum in the New Jersey Coastal Plain: Toward understanding the effects of global warming in a shelf environment. *Paleoceanography* **27**, PA4210 (2012).

15. Kim, J.-H. *et al.* New indices and calibrations derived from the distribution of crenarchaeal isoprenoid tetraether lipids: Implications for past sea surface temperature reconstructions. *Geochimica et Cosmochimica Acta* **74**, 4639–4654 (2010).

16. Schouten, S. *et al.* Extremely high sea-surface temperatures at low latitudes during the middle Cretaceous as revealed by archaeal membrane lipids. *Geology* **31**, 1069–1072 (2003).
