# Supplementary material for: Archaeal lipid biomarker constraints on the Paleocene-Eocene carbon isotope excursion
Source: Nat Commun. 2019 Oct 4;10:4519. doi: 10.1038/s41467-019-12553-3 (PMC6778145; doi:10.1038/s41467-019-12553-3)
Supplement: Supplementary file 3 — Description of Additional Supplementary files [file 41467_2019_12553_MOESM3_ESM.pdf]

Description of Additional Supplementary Files

File Name: Supplementary Data 1

Description: Compilation of estimates of carbon cycle and climate parameters for the Paleocene-Eocene carbon isotope excursion.

File Name: Supplementary Data 2

Description: Carbon isotope ratios and F2/F1 size ratios of crenarchaeol (cren) for sites IODP302 (Arctic Ocean), ODP174AX Ancora (New Jersey shelf), and ODP1172D (Tasman Sea). b.d.: below detection. mcd: meters composite depth. CSF: core depth below seafloor.

File Name: Supplementary Data 3

Description: Peak areas of isoprenoid and branched GDGTs as well as GDGT index and calculated BIT, TEX86, and sea surface temperature (SST) values for site ODP174AX Ancora (New Jersey shelf). GDGT-1a, GDGT-2a, and GDGT-3a are minor isomers eluting before the main peaks. Raw TEX86 values were corrected as described in the manuscript.

File Name: Supplementary Data 4

Description: Content and carbon isotope ratios of total organic carbon (TOC) as well as carbonate content calculated from weight loss following sample acidification for sites ODP174AX Ancora (New Jersey shelf) and IODP 302 (Arctic Ocean).
